# Localized Bodily Movements: Their Value as an Aid in the Treatment of Tuberculosis

**Published:** 1866-12

**Authors:** Robt. N. Tooker

**Affiliations:** Richmond, Ind.


### CHICAGO MEDICAL JOURNAL.

Vol. XXIII. DECEMBER, 1866. No. 12.

### ORIGINAL CONTRIBUTIONS.

*Localized Bodily Movements: Their value as an aid in the treatment of Tuberculosis.* By Robt. N. Tooker, M. D., Richmond, Ind.

In the August number of the *New York Medical Journal* is a review of a work just issued from the London press, by Stephen Henry Bigg, on the subject of "Localized Movements in the Treatment of Deformities and Chronic Diseases."

In that article the reviewer seems very favorably impressed with the theory of the practicability and usefulness of the author's system of practice. The idea of influencing the muscular system, and through it, the nervous and sanguineous, by means of external manipulations, is neither new nor confined to European practitioners. The treatment of paralysis by such forms of exercise as tend to stimulate nutrition in the cold and useless limbs, has long been a favorite adjunct of treatment with the more successful practitioners.

So also in certain forms of dyspepsia, and in nervous disorders, "fresh air and plenty of exercise" has been considered a *sine qua non* of cure. Indeed, the value and sphere of these "aids" has been found so great, that there are not a few in the profession who would more willingly dispense with medicine-case formulary than with them, in the treatment of chronic diseases.

The fact, however, that charlatans and quacks have, from time to time, usurped control of the idea of using bodily movements in the cure of disease, has led physicians, as a general thing, to look upon them with suspicion. Yet physicians, no less than the public, have stood amazed at the results often achieved by these same pretenders, who have cured, by "laying on of hands," and by various mysterious passes, cases that have baffled sound learning and large experience. So often is this witnessed, that, doubtless, there are many who, like the writer, have been forced to ask the question, "Can we not, as regularly educated and authorized practitioners of medicine, make use of whatever is proven to be good, without falling, in the estimation of our compeers, into the mire of quackery and empiricism?"

I propose, in this present paper, to give a condensed statement of what, from a somewhat extended practice during the past few years, and a very extended research among the writings of standard authors, I have been led to adopt as the correct theory of the causes and treatment of pulmonary tuberculosis.

The importance of the subject cannot be over-estimated. The mortality report of Cincinnati for the month of August last, shows *forty-four* deaths by consumption, which is more than *treble the number by any other disease excepting cholera*, and this, at a season of the year when febrile and bilious diseases are especially prevalent. The thousands who die every year by the hand of this fell destroyer in spite of the "best medical talent," and the accumulated experience which two thousand years have given us, are sufficient proof that there needs to be a better notion of the malady itself and of the appropriate remedy therefor than is generally entertained regarding them.

I shall not waste time and space here in rehearsing symptoms with which every one is familiar.

I proceed immediately to the discussion of the causes upon which the disease depends, and the conditions which are incident to it.

I advance the proposition, then, that whether the cachexia be hereditary or acquired, the disease itself is essentially and pre-eminently dependent on *vitiated nutrition*. In a disturbance of this grand function lies the prime cause of the disease, and those who seek in the lungs alone for either causes or effects will go wide of the mark. Dr. Thompson, chief physician to the London Hospital for consumption, who is a strong advocate of the virtues of cod oil, explains its mode of action by assuming that it assists in the manufacture of the blood corpuscles which are notoriously deficient in the blood of consumptives. Prof. Tanner says, "Tuberculosis is a blood disease, of which tubercle or tuberculous matter is the specific product." And when he speaks of the treatment, he says, emphatically, "when tubercles have become developed, we must first of all *endeavor to improve the general nutrition.*" Dr. Chambers says, in his late work on "The Renewal of Life:" "It is by aid of the digestive organs alone that consumption can be curable."

Medicines addressed to other parts may by useful sometimes indirectly, but they more commonly impede the recovery; whereas aid judiciously given in this quarter is always beneficial and usually successful. *We must leave the respiratory organs alone and direct our thoughts to the organs of nutrition, which will receive with thankfulness and return with interest any care we bestow upon them.* Authorities might be multiplied, if these were not sufficient to prove the assertion that it is not the lungs which are at fault, but other organs performing different functions. It is rather digestion, assimilation and absorption, in a word, "nutrition." The prevalent practice, therefore, of doctoring the lungs with expectorants and nauseants, either in the form of inhalations or of draughts internally administered, cannot be too strongly deprecated.

The lungs are but suffering the effects of causes foreign and remote, and it is only by correcting the causes of any trouble that we can expect to cure.

No wonder the efforts to cure consumption have met with failure, when the tubercles already formed have received the sole attention, and the tendency to new formations has been disregarded, or, at most, treated as of secondary importance. The enormous amount of lung surface available for ærating purposes, render it possible for large portions of this surface to suffer destruction without serious consequences. I believe that five-eighths of the lung tissue may be totally destroyed or rendered useless, without shortening life or materially impairing health, *provided the remaining three-eighths* remain intact. It is only when there is not enough well surface left to carry on the ærating process, that nature succumbs to the disease. So that even after large portions of the lungs have been involved in the destroying march of the disease, if we can arrest the further deposit of the tuberculous matter, we may safely assume that nature will adapt herself to the new condition of things, and so far as life and health are concerned, a cure will be effected. To show that the disease is not confined to the lungs, the fact is an established one, that when deposits of tuberculous matter are found there, (and sometimes when they are not,) they are also found in the liver, the spleen, the kidneys; in the various glands, the sheath and coverings of muscles, and even in the bones, as in Pott's disease of the spine.

The disease is then general and constitutional. Nutrition is at fault—and it is to nutrition that we should direct our attention and all our remedial efforts. Before speaking of the means at our disposal for correcting this evil tendency on the part of the constitution, which we have seen to be the cause of all this trouble, I wish to speak of the action of oxygen on the organism, since very much of the value which will be given to the treatment soon to be advocated, will depend directly on the uses which we assign to this element. The opinion is a very prevalent one that the consumptive patient is dying for want of oxygen; that the blood is but imperfectly oxydized, and that any agent that will supply this deficiency, will in so far be pregnant with good. With this idea, almost every *oxide* that chemistry could suggest has been tried, and abandoned for some new preparation which promised to furnish in a better form this indispensable element. With the same end in view, many excellent practitioners have advocated the use of inhaling tubes, spirometers and chest-expanders, which, by enlarging the capacity of the lungs, would increase directly the amount of oxygen inspired. Granting for a moment that the *tissues* do stand in need of oxygen—for it surely is not simply to pass into the stomach or the lungs and out again—it is difficult to see how this oxygen can ever reach the tissues in the absence of the blood corpuscles which are the oxygen carriers, or when these blood corpuscles are as largely increased in number as they are in the blood of consumptives. But so far from there being this need for more oxygen, I shall endeavor to show that the reverse is the case; that the system needs less rather than more, and that the use of inhaling tubes and spirometers is not only reprehensible, from the fact that this forcible insufflation is liable to break down the texture of the lungs by over-distension of the air cells, as has happened in numerous instances, but for the reason that an increased volume of air is not desirable but positively detrimental.

If the consumptive does need more air, it is because he requires more of the oxygen which the air contains, for, although the atmosphere contains other elements, neither chemistry nor physiology point out their uses. They are not as yet understood. But chemistry and physiology teach us that the process of oxydation is, in part at least, a *combustion process*, or, if not literally and strictly so, it may, for all practical purposes, be so considered. For although it is clearly proven that the inspired oxygen does not combine directly with the carbon of the blood while in the lungs, and that the production of blood does not take place here, as was formerly supposed—thus making the lungs the fireplace of the system—yet it is admitted on all hands that the oxygen does serve the double purpose of a vitalizer and a devitalizer or decomposer, and it is more than probable that this latter is the greater and more important part of its function. In the process of waste which the several tissues are constantly undergoing, and by which their particles are constantly becoming useless and burdensome, it is the function of the oxygen to so combine with this debris as to form new and soluble compounds, which the various excretory organs can eliminate from the system, to make room for the newly vitalized matter which are to supply the deficiency. In health this oxydizing process goes on but slowly, because the tissues have, by virtue of the vital principle which pervades them, something of a resistant power against the oxygen, which otherwise would quickly destroy them, and they thus preserve their integrity for a longer period than when the system is depressed by disease, and the resistant power is in consequence lowered. This is illustrated in the phenomena of external injuries. When the tegumentary tissues are so injured by violence or disease as to lose this power of resistance to the action of oxygen, then ulceration or slough takes place, and although the action of the oxygen contained in the atmospheric air is invigorating and beneficial to these tissues in health, yet when their vitality is thus lowered, exposure to it kills them still further, and may soon alone prove fatal, as we see in cases of extensive burns. This illustrates the utility of the practice in smallpox of painting the pocks over with collodion or caoutchouc, to preserve them from the air, and thus prevent the deep ulcerations which would otherwise ensue.

It must be borne in mind that the condition of inflamed tissues is materially changed from that of health, and their natural susceptibilities become from this cause so morbidly increased that even their natural stimuli cannot be tolerated, but if allowed to act, become additional and powerful sources of mischief. The natural stimuli of the eye, the ear, the stomach and the bladder, are grateful during the normal state of these organs; but when inflammation occurs, the smallest pencil of light becomes intensely painful to the eye; noises to the ear; food and drinks to the stomach, and urine to the bladder. The cells which compose these inflamed tissues, unable to retain their integrity, by having their sources of nourishment cut off, by reason of the congestion, and also deprived of their nerve stimulus, degenerate into pus. The body deprived of the vital principle speedily decays—that is, *becomes oxydized.* Living, healthy tissues, either by virtue of this vital principle, or some equally powerful cause, do not yield themselves thus readily to the action of oxygen, but once lower their resistant power, and destruction is the result. The universal wholesomeness of fresh air is probably a mistaken notion. It is bad for inflamed skins; it is bad for abscesses; it is bad for wounds: *is it good for inflamed lungs?*

With softening tubercles in the lung substance, and the neighboring parts inflamed and irritable, the more oxygen is inspired, the more certain, rapid and widespread the destruction must be. But not only here, but, by the very nature of the disease, the body is being too rapidly consumed; it is *consumption*, not figuratively but literally. The waste of the tissues exceeds the repair; disintegration of structure predominates over the process of reconstruction. There is inanition or starvation, though a full supply of food is taken into the stomach, because this food is either misapplied, or assimilation and absorption being faulty, the food never becomes blood, and hence never reaches the tissues.

In Dr. Gardner's treatise on gout, are some very interesting and important experiments concerning the action of oxygen on the blood, and they are so *apropos*, I cannot avoid mentioning them in this connection. In these experiments, performed on rabbits, it was found that after pure oxygen had been respired for half an hour, the quantity of albumen and blood corpuscles was materially diminished, while the quantity of fibrin was largely increased. But the two former elements are the ones which make the blood, the nutrient fluid of the body. The corpuscles are indeed the most important of all its ingredients, and next in importance comes the albumen. And these are both diminished as the quantity of inspired oxygen is increased. As regards the fibrin, Dr. Smith, the Am. Ed. of Carpenter's Physiology, says, in commenting on the views held by Prof. C. relative to it:

"This product, (fibrin,) it is maintained by many, in opposition to the views here stated, is to be traced to the process of disintegration and waste that is going on constantly in the system, and that opinion gains support from the fact that in diseases characterized by rapidity of capillary circulation and emaciation, such as phthisis, anemia and acute inflammation, *this material is strikingly increased.*" The diminution of the blood corpuscles in these same diseased conditions, is a well authorized fact. If, therefore, an augmented inspiration of oxygen tends, as these experiments go to show, to increase the product of waste—which in consumption are already too great—while at the same time it diminishes those elements which by their presence constitute the life and usefulness of the blood, there is surely something wrong in that practice which consists in *an increased amount of oxygen*, be it given in the form of the iodides, the chlorides, inhaling tubes, or the "compressed air bath." Such a practice can only be supported on Hahnemannic grounds—"Similia similibus curantur."

I believe, therefore, that too much importance has been ascribed to the vitalizing power of inspired oxygen, and not enough to the elimination of carbonic acid and the volatile organic matter which result from every functional manifestation. It is true that an animal will not live for any considerable time in an atmosphere of pure hydrogen—though the elimination of of carbonic acid is in no manner impeded. But, on the other hand, an animal made to breathe an atmosphere of carbonic acid alone will die almost immediately, as the experiment of Spallanzani and W. Edwards prove. The experiments of Claude Bernard are also in point. He mingled fifty per cent. of oxygen with fifty per cent. of carbonic acid, and found that warm-blooded animals were suffocated in it in spite of there being more than double the quantity of oxygen present in the ordinary atmosphere. The inference of Bernard is doubtless the true one: that the inability of the atmosphere to sustain life, is due to the obstacle it offers to the exhalation of carbonic acid. The blood cannot free itself of the carbonic acid it contains, and cannot therefore become *arterialized*, notwithstanding the presence of so much oxygen, because the conditions necessary to exchange are absent. Other experiments of Bernard's are still more to the point. He tied a dog's head in a bag, which would in a certain time produce suffocation, and he found that this period was by no means retarded when he *injected oxygen into the arteries*. On the other hand, he injected large quantities of carbonic acid into the veins and arteries, and under the skin of rabbits, and so long as the lungs were free to act, no noxious effect ensued. The more of this element he injected into the system, the greater the quantity exhaled by the lungs. And again, he found that a bird about to expire in vitiated air, could be readily brought to life again by the introduction of potash, which would absorb the carbonic acid—showing that it was from the presence of this carbonic acid that respiration was impeded.

There are a few final considerations which go to sustain the views which I have presented, and I desire to briefly state them before passing to the treatment.

(1st.) In no other satisfactory way can we explain the beneficial results which so commonly follow the use of alcohol and oils, when administered in this disease. The former, at least, does not enter into the system as one of its component elements, nor does it remain in the organism in any known form. It does not supply any deficiency then directly, and yet, ample evidence could be adduced to prove its value as a remedy. The only way in which we can account for the fact is by supposing that it acts as a conservator of tissue. In keeping up the heat of the body, it supplies the place of food and tissue.

(2d.) In hot climates where the air is highly rarified, a less volume of oxygen is taken into the lungs at each inspiration, than in colder climates, to enter into combination with the carbon of the system. On this account we observe a greater prevalency of the liver and bilious affections in torrid than in temperate latitudes. Unless care be taken to avoid liquors and articles of food which produce a large amount of cárbon, a greater quantity of other elements will be assimilated than the inspired oxygen can decompose. Thus an excess of carbon and hydrogen is retained, relaxing and enfeebling the nervous and muscular tissues, so that from slight exciting causes, diseases of a bilious and congestive nature are originated. But we find here a marked exemption from tuberculous disease.

(3d.) The fact is becoming better and better established each year that consumptives do better in these hot climates than in colder ones, and the practice of sending patients, affected with tubercles, to northern latitudes, is being rapidly abandoned, as experience proves that such a practice is detrimental rather than beneficial.

(4th.) Corpulent people are proverbial, as a class, for narrow, contracted chests, while, as a class, they are remarkably exempt from consumption. Unless there be some additional fault in the constitution, narrow-chested persons are not proven to be more disposed to consumption than are those with larger lung capacity.

In accordance with the foregoing views, our curative efforts should be directed to the accomplishment of these three objects: First, to limit the quantity of oxygen inspired; second, to supply, in a concentrated form, carbonaceous foods, which this oxygen will act upon instead of upon the carbon of the system; and third, stimulate the renewal of the tissues, and increase their resistant power toward the oxygen which is destroying them.

As regards the accomplishment of the first object, Thomas King Chambers, who, from his position and experience, is competent authority, says: "I find that few things contribute to the ease and recovery of my patients so much as limiting the supply of oxygen in the atmosphere *by saturating it with watery vapor.* I refer here not only to pneumonia, but to all affections of the breathing apparatus.* This can be accomplished by the ordinary process of evaporation, or still better, by the use of the *atomizer*, which may be allowed to throw a small jet of steam into the room the greater part of the time. The second object can be best accomplished by the use of cod liver oil and whisky, or some substitute for them.

I wish to speak more particularly of the third part of the treatment, whose object can be secured by the judicious use of exercise or bodily movement, since they tend directly to stimulate and strengthen both the nervous and muscular tissues. They also help on digestion, so that more food is assimilated and brought into the circulation for the oxygen to act upon.

We must correct the balance between waste and renewal, and nothing is so capable of doing this as muscular contractions, cautiously employed. Exercise has long been recommended and employed in this disease, and has often proved highly salutary, though the exercise has been of the simplest and most unsystematic kind. But in general, simply to "take exercise," may neither improve health nor strength, it may injure both. Weakness no less than strength may follow the

* "Renewal of Life," page 91.

employment of any function. The healthiest organism may be, and often is, weakened and diseased by exercise too violent or too prolonged, and surely, much more care should be taken to regulate the kind and amount, when exercise is prescribed for medicinal ends, and to be taken by a body already enfeebled by disease.

The good and eminent Dr. Rush, the father of American medicine, appreciating the truth of this, wrote, "That physician does not err more who advises a patient to take physic without specifying its kind and quantity, than the physician who advises a patient in consumption to use exercise, without specifying its species and degrees. From the neglect of this direction we often find consumptive patients injured instead of being relieved by exercise, which, if used with judgment, might have been attended with the happiest results."

Probably in no other disease is the matter of the *appropriate* exercise of more importance.

The utmost care should be taken to prevent congesting the lungs by such exercises as cause their effects to center in the region of the chest. All violent active movements of the arms, such as swinging dumb bells or heavy clubs, or indeed any violent effort which will hurry the breathing, should be scrupulously avoided. The lighter calisthenic exercises, like those employed in Dio Lewis' Light Gymnastics, are much preferable. But even these may be productive of harm, especially if the disease be considerably advanced, and the powers consequently weak. In these cases, only *passive* movements, or those partially active, are admissible. These will require an assistant, but may be given by any one in attendance upon the invalid, under the doctor's direction. The movements themselves consist in rubrings, or ordinary frictions; rollings of the muscles, effected by placing a limb between the hands and rubbing rapidly *across* the axis of the limb; spatting the muscles with the palm of the hand, or still better, *hacking* rapidly over the muscles with the edge of the hand. While the patient lies passive the operator may flex the limbs in various ways, causing alternate contractions and expansions of the muscular fibres, the patient meantime, if able, offering slight resistance to these manipulations.

The greatest care must be exercised at first, and indeed all the time, to adjust these efforts to the patient's strength. *No effort should be carried to fatigue.* The nervous forces in particular must be shielded and strengthened, not exhausted. Hence the value of the passive movements. These irritate the muscles and regulate the circulation without irritating the nerves or wasting their energies.

I have seen the quick, fluttering pulse of hectic, calm down and become regular, and many distressing symptoms disappear temporarily, under an hour's judicious handling in this way, and permanent benefit follow, by persistence in the treatment. After continuing these manipulations for a few minutes, the patient should rest perfectly quiet for a time, after which they may be again resumed.

I do not deem it necessary to be more explicit. An endless number of movements may be devised by the physician, varied as the cases vary.

The main idea being, always to get the greater number of muscular contractions possible, commensurate with the patient's strength, always stopping short of fatigue.

