# Errata

**Published:** 1867-01

**Authors:** 


### ERRATA.

In the article on Consumption, by Dr. Tooker, published in our last issue, page 543, third line, read *diminished* instead of *increased;* and in the twenty-third line upon the same page, the word *heat* should be substituted for the word *blood.*

